# Comparison of the effects of childhood demographic characteristics on physical activity during adulthood across 22 countries

**DOI:** 10.1186/s12889-025-23430-8

**Published:** 2025-07-05

**Authors:** Chung Gun Lee, Elizabeth Kwon, Jason Paltzer, Chikwuemeka N. Okafor, Tyler J. VanderWeele, Byron R. Johnson, Junhye Kwon

**Affiliations:** 1 Department of Physical Education, College of Education, Seoul National University, 71-1, Seoul, 08826 South Korea; 2 Institute of Sport Science, Seoul National University, Seoul, 1 Gwanak-Ro, Gwanak-Gu, Seoul, 08826 South Korea; 3 Department of Public Health Baylor University, Waco, TX USA; 4 The Meros Center, Wisconsin Lutheran College, Milwaukee, WI USA; 5 Department of Medicine, Division of Infectious Diseases, Long School of Medicine, University of Texas Health Science Center San Antonio, Antonio, TX USA; 6 Departments of Epidemiology and Biostatistics, Harvard T.H. Chan School of Public Health, Boston, MA USA; 7 Harvard University, Human Flourishing Program, Cambridge, MA USA; 8 Institute for Studies of Religion, Baylor University, Waco, TX USA; 9 School of Public Policy, Pepperdine University, Malibu, CA USA

**Keywords:** Physical activity, Childhood, Demographic factors, Country differences, Meta-analysis

## Abstract

**Background:**

This study examines the impact of childhood demographic characteristics, such as socioeconomic status, health and well-being status, family dynamics and relationships, and religious and spiritual status, on physical activity levels in adulthood across 22 countries.

**Methods:**

We analyzed data from the Global Flourishing Study (GFS), which includes 202,898 participants across 22 countries. The study examined whether childhood characteristics predict physical activity levels in adulthood and whether these relationships differ by country.

**Results:**

The relationships between childhood predictors and physical activity in adulthood varied across countries. However, the overall results showed that self-rated health and religious service attendance during childhood, as well as age and gender, were significantly associated with physical activity levels in adulthood.

**Conclusions:**

The findings highlight the critical role of childhood self-rated health and religious involvement in shaping physical activity habits in adulthood across 22 countries. These results lay the groundwork for future GFS data (2024 through 2027) to guide interventions promoting physical activity among adults globally.

**Supplementary Information:**

The online version contains supplementary material available at 10.1186/s12889-025-23430-8.

## Introduction

Regular physical activity is a crucial modifiable health behavior linked to a protective association with chronic diseases and promoting physical health and mental well-being [1]. Although the health benefits of regularly performing physical activity are widely acknowledged [2] and engaging in regular physical activity over time is strongly associated with reduced all-cause mortality [3], physical inactivity poses a significant public health burden [4]. Developing and implementing policies and programs aimed at effectively enhancing people’s physical activity is, therefore, a critical area of public health. The key step in developing and implementing physical activity interventions is to understand the determinants of physical activity [5, 6].

Similar to numerous health-related behaviors or outcomes, evidence from previous studies indicates that physical activity is associated with various demographic characteristics, such as socioeconomic status [7–9], family dynamics and relationships [10–12], health and well-being status [13, 14], and religious and spiritual status [15]. Despite inconsistencies in findings and debates over which indicators of socioeconomic status are more strongly associated with physical activity [8, 9], evidence indicates that individuals from less advantaged socioeconomic backgrounds participate less frequently in physical activity compared to their more advantaged counterparts among both youth [7] and adults [8, 9]. This disparity might be influenced by reduced access to physical activity related facilities and fewer opportunities for engaging in physical activity. Some potential mechanisms in the association between family dynamics and physical activity have also been proposed. Family dynamics and relationships have been demonstrated to play a crucial role in promoting mental well-being, which may, in turn, encourage self-care behaviors such as exercise and physical activity. [16, 17]. Improved family relationships may also enhance self-esteem, potentially aiding individuals in adopting healthy behaviors and avoiding unhealthy lifestyle habits, such as sedentary behavior [18, 19]. Few studies have investigated the relationship between self-rated health and physical activity. The evidence from these studies indicates that recreational physical activity positively influences subjective health assessments, whereas no correlation was found between physical workload and self-perceived health status. These results may suggest that heavy physical labor could increase the risk of cardiovascular and other chronic diseases, or even mortality. A recent review on the relationship between religion and physical activity indicates that religion and spirituality are positively associated with engaging in moderate to vigorous physical activity among various populations [15]. Because regular physical activity provides similar health benefits related to social interactions, physical health, and psychological well-being, physically active individuals may be more inclined to participate in spiritual and religious practices.

Compared to more temporally proximate relationships between these demographic characteristics and physical activity behavior, evidence linking childhood characteristics and experiences to physical activity in adulthood is relatively scarce. One review study examined the hypothesis that lower socioeconomic status during childhood is related to less frequent leisure-time physical activity in adulthood. Out of fifty-five studies, evidence supported the hypothesis in twenty-two studies, while no association is found in thirteen studies. Accounting for one’s own socioeconomic status in adulthood partly weakened this association. Moreover, there was stronger evidence of an association in British cohorts compared to Scandinavian cohorts, underscoring the significance of cross-country comparisons in this association [20]. To our knowledge, there is only one study that has examined the association between the relationship with parents during early life and physical activity during adulthood. The study found that the relationship with parents in adolescence significantly influenced sport participation in young adulthood, specifically among female participants [21]. These results suggest that associations between childhood demographic features and adult physical activity may typically exhibit significant magnitude and may not be entirely explained by the continuity of each demographic feature from childhood to adulthood. It is plausible that adult physical activity reflects early life demographic characteristics, as different stages in an individual's life course are closely interconnected. Significant conditions and events in one stage of an individual’s life may influence subsequent stages, shaping their trajectory of conditions and events over time [22]. If this holds true, it will be essential to uncover the mechanisms that explain how various demographic characteristics in early life influence physical activity behavior later in life.

The primary objective of this study is to examine the potential role of childhood demographic characteristics, such as socioeconomic status, family dynamics and relationships, health and well-being status, and religious and spiritual status, in influencing physical activity levels during adulthood. Given that previous studies have primarily focused on developed countries, we explore how these childhood demographic factors are associated with adult physical activity levels across 22 countries worldwide in the Global Flourishing Study, with large sample sizes and nationally representative samples. Monitoring global trends in physical activity levels and their determinants is crucial for laying the groundwork for establishing effective national and international interventions and policies.

## Methods

The description of the methods below has been adapted from VanderWeele et al. (2024). Further methodological detail is available elsewhere [23–28].

### Study population

We used data from the Global Flourishing Study (GFS), which investigates the distribution and determinants of well-being across a sample of 202,898 individuals from 22 geographically and culturally diverse countries. The first wave of GFS collected nationally representative data across Argentina, Australia, Brazil, Egypt, Germany, Hong Kong (Special Administrative Region of China, including mainland China from 2024 onwards), India, Indonesia, Israel, Japan, Kenya, Mexico, Nigeria, the Philippines, Poland, South Africa, Spain, Sweden, Tanzania, Turkey, the United Kingdom, and the United States. These countries were selected to ensure broad coverage of the global population, encompassing geographical, cultural, and religious diversity. Data collection by Gallup Inc primarily occurred in 2023, with initial phases in some regions starting in 2022, varying by country. Detailed information on the sampling design for achieving national representativeness is available elsewhere. Data from the study are publicly accessible through the Center for Open Science (https://www.cos.io/gfs). The translation process followed the TRAPD model (translation, review, adjudication, pretesting, and documentation) for cross-cultural survey research (ccsg.isr.umich.edu/chapters/translation/overview). Additional details on methodology, survey development, and translations can be found in the GFS Questionnaire Development Report [23], GFS Methodology [27], GFS Codebook [29], and GFS Translations documents [30, 31].

### Measures

#### Physical activity

Physical activity was assessed by asking “On how many days did you exercise or engage in vigorous physical activities for 30 min or more in the past week?” Response options ranged from: 0 = 0 days to 7 = 7 days/every day. Higher scores indicated greater physical activity participation.

#### Childhood predictors

We evaluated 11 predictors that were assessed at baseline. Each predictor is numbered below (#1-#11), and organized into five categories (A-E).

A) Demographic predictors*,* included: #1) Age/Year of Birth (“What is your age? Please enter your age below.” Age was classified as 18-24, 25-29, 30-39, 40-49, 50-59, 60-69, 70-79, and 80+), #2) Gender (“What is your gender?” Response options were: Male, Female, or Other), and #3) Immigration status (“Were you born in this country, or not?” Response options were: Yes or No). Race/ethnicity was also assessed, but not analyzed as a childhood predictor because race/ethnicity differed across the 22 countries.
B) Socioeconomic predictors, included: #4) Subjective financial status of family growing up: (“Which one of these phrases comes closest to your own feelings about your family's household income when you were growing up, such as when YOU were around 12 years old?” Response options were: Lived comfortably, Got by, Found it difficult, or Found it very difficult).
C) Family dynamics and relationship predictors, included: #5) Parent marital status / Family structure (“Were your parents married to each other when YOU were around 12 years old?” Response options were: Married, Divorced, Never married, One or both had died), #6) Relationship with mother (“Please think about your relationship with your mother when you were growing up. In general, would you say that relationship was…” Response options were: Very good, Somewhat good, Somewhat bad, or Very bad?” Responses were dichotomized to Very/Somewhat good versus Very/Somewhat bad), #7) Relationship with father (An analogous question and answers were used to assess relationship with father). “Does not apply” was treated as a dichotomous control variable for respondents who did not have a mother or father due to death or absence, #8) Felt like an outsider in family growing up (“When you were growing up, did you feel like an outsider in your family?” Response options were: Yes or No), and #9) Abuse (“Were you ever physically or sexually abused when you were growing up?” Response options were: Yes or No).
D) Health and well-being predictors, included: #10) Self-rated health growing up (“In general, how was your health when you were growing up?” Response options were: Excellent, Very good, Good, Fair, or Poor).
E) Religious and spiritual predictors, included: #11) Age 12 religious service attendance (“How often did YOU attend religious services or worship at a temple, mosque, shrine, church, or other religious building when YOU were around 12 years old?” Response options were: At least once/week, One-to-three times/month, Less than once/month, or Never. Childhood religious tradition/affiliation had response categories of Christianity, Islam, Hinduism, Buddhism, Judaism, Sikhism, Baha’i, Jainism, Shinto, Taoism, Confucianism, Primal/Animist/Folk religion, Spiritism, African-Derived, some other religion, or no religion/atheist/agnostic; precise response categories varied by country [30]. This latter factor was not analyzed as a childhood predictor because it differed across the 22 countries.

### Statistical analysis

Descriptive statistics for the weighted sample, adjusted to reflect national representation within each country, were calculated across various childhood demographic categories. Subsequently, within each country, a weighted linear regression model was fitted, adjusting for complex survey design, to examine the association between physical activity and all childhood predictor variables simultaneously. In the primary analyses, random-effects meta-analyses were performed to synthesize regression coefficients [32, 33], providing corresponding confidence intervals, 95% prediction intervals (lower and upper limits), estimates of heterogeneity (τ), and I^2^ statistics to assess variability across countries within each demographic category [34]. Forest plots displaying these estimates are available in the supplementary materials.

Religious affiliation/tradition and race/ethnicity were included as control variables within countries when available; however, due to differences in responses across countries, their regression coefficients were excluded from the meta-analysis results. All meta-analytical procedures were conducted in R (R Core Team, 2024) using the metafor package [35]. For each country, a global test was performed to assess the association between groups of childhood predictors and physical activity outcomes, and a pooled p-value [36] across countries was calculated and reported to assess evidence for associations at the country level. Bonferroni-corrected significance thresholds were applied to account for multiple comparisons across childhood demographic variables [37, 38].

Additionally, for each predictor variable, E-values were calculated to assess the robustness of observed associations against potential unmeasured confounding. An E-value represents the minimum strength of association that an unmeasured confounder would need to have with both the predictor and the outcome, beyond measured covariates, to fully explain away the observed association [39]. As a supplementary analysis, meta-analyses weighted by population size were also conducted.

All analyses were pre-registered at the Center for Open Science before accessing the data, with only minor modifications made to regression models later due to multicollinearity issues (https://osf.io/yrn8h). The complete analysis code is available in a publicly accessible online repository [25].

### Missing data

Missing data for all study variables were addressed using multivariate imputation by chained equations, generating five imputed datasets for analysis [40–42]. To accommodate differences in the measurement of certain variables across countries (such as religious affiliation/tradition and race/ethnicity), imputations were performed separately within each country. This country-specific imputation strategy ensured that the models appropriately reflected national contexts and assessment procedures. Additionally, sampling weights were incorporated into the imputation models to account for missingness that might be associated with the probability of selection into the study.

### Accounting for complex sampling design

Different sampling strategies were employed across countries in the GFS, depending on the availability of existing panels and specific recruitment requirements [27]. All analyses incorporated elements of the complex survey design, including the use of weights, primary sampling units, and stratification. Further methodological information, particularly regarding the consideration of the complex sampling design, can be found elsewhere [26].

## Results

### Descriptive statistics

Table 1 presents the distribution of descriptive statistics. Age/birth year spanned the entire adulthood (18–80 +). The gender distribution was almost even, with 51% female, 49% male, along with a small number of other genders. The majority of participants were native-born (94%) and had married parents (75%). Religious service attendance in childhood varied, with most attending weekly or more frequently (41%), some never attending (23%), others attending less than once a month (18%), and the rest attending one to three times a month (16%).

**Table 1:** Nationally-representative descriptive statistics of the observed sample (*N* = 202,898)

Variable	*N* (%)
Relationship with mother	
Very good	127836 (63%)
Somewhat good	52439 (26%)
Somewhat bad	11060 (5%)
Very bad	4642 (2%)
Does not apply	5965 (3%)
Missing	956 (0%)
Relationships with father	
Very good	107742 (53%)
Somewhat good	55714 (27%)
Somewhat bad	15807 (8%)
Very bad	8278 (4%)
Does not apply	13985 (7%)
Missing	1372 (1%)
Parent marital status	
Parents married	152001 (75%)
Divorced	17726 (9%)
Single, never married	15534 (8%)
One or both parents had died	7794 (4%)
Missing	9843 (5%)
Subjective financial status of family growing up	
Lived comfortably	70861 (35%)
Got by	82905 (41%)
Found it difficult	35852 (18%)
Found it very difficult	12606 (6%)
Missing	674 (0%)
Abuse	
Yes	29139 (14%)
No	167279 (82%)
Missing	6479 (3%)
Outsider growing up	
Yes	28732 (14%)
No	170577 (84%)
Missing	3589 (2%)
Self-rated health growing up	
Excellent	67121 (33%)
Very good	63086 (31%)
Good	47378 (23%)
Fair	19877 (10%)
Poor	4906 (2%)
Missing	530 (0%)
Immigration status	
Born in this country	190998 (94%)
Born in another country	9791 (5%)
Missing	2110 (1%)
Age 12 religious service attendance	
At least 1/week	83237 (41%)
1-3/month	33308 (16%)
<1/ month	36928 (18%)
Never	47445 (23%)
Missing	1980 (1%)
Year of birth	
1998-2005; age: 18-24	27007 (13%)
1993-1998; age 25-29	20700 (10%)
1983-1993; age 30-39	40256 (20%)
1973-1983; age 40-49	34464 (17%)
1963-1973; age 50-59	31793 (16%)
1953-1963; age 60-69	27763 (14%)
1943-1953; age 70-79	16776 (8%)
1943 or earlier; 80 or older	4119 (2%)
Missing	20 (0%)
Gender	
Male	98411 (49%)
Female	103488 (51%)
Other	602 (0%)
Missing	397 (0%)
Country	
Argentina	6724 (3%)
Australia	3844 (2%)
Brazil	13204 (7%)
Egypt	4729 (2%)
Germany	9506 (5%)
Hong Kong	3012 (1%)
India	12765 (6%)
Indonesia	6992 (3%)
Israel	3669 (2%)
Japan	20543 (10%)
Kenya	11389 (6%)
Mexico	5776 (3%)
Nigeria	6827 (3%)
Philippines	5292 (3%)
Poland	10389 (5%)
South Africa	2651 (1%)
Spain	6290 (3%)
Sweden	15068 (7%)
Tanzania	9075 (4%)
Turkey	1473 (1%)
United Kingdom	5368 (3%)
United States	38312 (19%)

*Country-specific descriptive statistics can be found in the Online Supplement

### Childhood predictors – meta-analytic results

Table 2 demonstrates associations between the 11 childhood variables and physical activity in adulthood. Several childhood predictors were associated with increased physical activity in adulthood, including: 1) experiencing excellent health growing up [β = 0.17, 95% CI: 0.07, 0.26], 2) growing up in a family with sufficient financial resources [β = 0.11, 95% CI: 0.05, 0.18], 3) attending religious services at age 12 at least once a week [β = 0.21, 95% CI: 0.11, 0.32] or one to three times a month [β = 0.17, 95% CI: 0.07, 0.27]. In terms of gender, female [β = −0.46, 95% CI: −0.61, −0.32] and individuals identifying as other [β = −0.69, 95% CI: −1.17, −0.22] engaged in less physical activity in adulthood compared to males.

**Table 2:** Random effects meta-analysis of regression of physical activity on childhood predictors

					Estimated Proportion of Effects by Threshold				
Variable	Category	Est	95% CI	SE	< −0.10	> 0.10	Heterogeneity (τ)	I^2	Global p-value
Relationship with mother	(Ref: Very bad/somewhat bad)								<.001**
	Very good/somewhat good	0.02	(−0.07,0.10)	0.04	0.18	0.23	0.12	37.8	
Relationship with father	(Ref: Very bad/somewhat bad)								<.001**
	Very good/somewhat good	0.03	(−0.02,0.08)	0.03	0.00	0.00	<.01ǂ	< 0.1ǂ	
Parent marital status	(Ref: Parents married)								<.001**
	Divorced	−0.04	(−0.09,0.02)	0.03	0.00	0.00	0.02	2.8	
	Single, never married	−0.08	(−0.16,0.00)	0.04	0.32	0.00	0.10	28.4	
	One or both parents had died	0.04	(−0.09,0.17)	0.07	0.23	0.36	0.22	55.4	
Subjective financial status of family growing up	(Ref: Got by)								<.001**
	Lived comfortably	0.11	(0.05,0.18)	0.03	0.05	0.55	0.12	64.9	
	Found it difficult	−0.04	(−0.11,0.02)	0.03	0.27	0.09	0.11	50.1	
	Found it very difficult	0.02	(−0.10,0.14)	0.06	0.23	0.36	0.20	53.1	
Abuse	(Ref: No)								<.001**
	Yes	−0.01	(−0.09,0.07)	0.04	0.33	0.19	0.13	54.8	
Outsider growing up	(Ref: No)								<.001**
	Yes	0.02	(−0.04,0.09)	0.03	0.05	0.18	0.09	36.1	
Self-rated health growing up	(Ref: Good)								<.001**
	Excellent	0.17	(0.07,0.26)	0.05	0.14	0.64	0.20	75.6	
	Very good	0.06	(−0.02,0.14)	0.04	0.09	0.32	0.15	66.4	
	Fair	−0.06	(−0.14,0.03)	0.04	0.32	0.05	0.13	43.7	
	Poor	−0.04	(−0.16,0.07)	0.06	0.18	0.00	0.08	8.0	
Immigration status	(Ref: Born in this country)								<.001**
	Born in another country	0.00	(−0.13,0.13)	0.07	0.23	0.32	0.20	53.5	
Age 12 religious service attendance	(Ref: Never)								<.001**
	At least 1/week	0.21	(0.11,0.32)	0.05	0.05	0.86	0.19	67.9	
	1–3/month	0.17	(0.07,0.27)	0.05	0.05	0.64	0.18	62.7	
	< 1/month	0.06	(−0.01,0.13)	0.04	0.09	0.23	0.10	41.7	
Year of birth	(Ref: 1998–2005; age 18–24)								<.001**
	1993–1998; age 25–29	−0.12	(−0.23,−0.01)	0.06	0.50	0.09	0.21	69.5	
	1983–1993; age 30–39	−0.15	(−0.28,−0.01)	0.07	0.59	0.09	0.30	84.8	
	1973–1983; age 40–49	−0.17	(−0.36,0.02)	0.10	0.50	0.23	0.43	91.0	
	1963–1973; age 50–59	−0.09	(−0.33,0.14)	0.12	0.45	0.36	0.54	93.2	
	1953–1963; age 60–69	−0.01	(−0.30,0.29)	0.15	0.50	0.41	0.68	94.2	
	1943–1953; age 70–79	−0.16	(−0.47,0.15)	0.16	0.64	0.27	0.68	91.8	
	1943 or earlier; age 80 +	−0.57	(−0.97,−0.16)	0.21	0.73	0.27	0.87	89.1	
Gender	(Ref: Male)								<.001**
	Female	−0.46	(−0.61,−0.32)	0.07	0.91	0.00	0.33	95.3	
	Other	−0.69	(−1.17,−0.22)	0.24	0.72	0.17	0.90	87.9	

^*^p <.05; **p <.004 (Bonferroni corrected threshold); ǂestimate of heterogeneity is likely unstable, please see our online supplement forest plots for more detail on heterogeneity of effects

### Country-level variation

The meta-analysis also revealed considerable heterogeneity in how early-life conditions impact physical activity in adulthood across the 22 countries. First, self-rated health during childhood showed high heterogeneity (τ = 0.20, I2 = 75.6% for experiencing very excellent health in childhood), indicating that the impact of childhood self-rated health on adulthood physical activity varied significantly by countries. Secondly, religious service attendance at age of 12 (attending one to three times a month: τ = 0.18, I2 = 62.7%; at least once a week: τ = 0.19, I2 = 67.9%) also showed substantial variability. This suggests that the frequency of the religious service attendance in childhood plays a crucial role in adult physical activity, but in ways that vary remarkably across countries. Finally, the effect of birth year exhibited high heterogeneity, which increased with specific age ranges (comparing 18–24 year-olds to 25–29 year-olds, τ = 0.21, I2 = 69.5 and 18–24 year-olds to 30–39 year-olds, τ = 0.30, I2 = 84.8, while, comparing 18–24 year-olds to 80 + year-olds, τ = 0.87, I2 = 89.1).

Certain childhood factors showed almost universal positive associations with adult physical activity across countries (i.e., excellent health growing up, financially comfortable in childhood, attending religious service at least weekly or one to three times a month), whereas others were nearly negative (i.e., age 25–29, 30–39, or 80 +, identifying as female or another gender). Other childhood variables, such as relationship with mother or father, parental marital status, immigration status, experiences of abuse, and feeling like an outsider when growing up showed mixed associations with physical activity across countries. Notably, Nigeria was the only country in which having a very good or somewhat good relationship with both the mother and the father—independently—was positively associated with adult physical activity, compared to those reporting poor relationships. In contrast, in countries such as the Philippines and Türkiye, only the relationship with the mother showed a significant positive association, while the relationship with the father did not.

Age/birth year exhibited substantial heterogeneous effect sizes across countries (Figures S21-27). In United States and Australia, the association between age and physical activity is negligible. In Japan and Indonesia, physical activity increases across age cohorts, whereas in Egypt and Poland, younger generations report greater physical activity.

The E-values indicated that several observed associations exhibited moderate robustness to the potential influence of unmeasured confounding factors (Table 3). A high E-value indicates that the observed effect size is more robust to unmeasured confounders, while a low E-value suggests that the observed effect size is more vulnerable to confounding. For instance, in the context of subjective financial status in childhood, an unmeasured confounder could explain away the observed association. Specifically, this confounder would need to be associated with both financial comfort and higher physical activity, each with risk ratios of 1.26, beyond the covariates already adjusted for. Weaker joint confounder associations, however, would not suffice. Additionally, to shift the confidence interval to encompass the null value, the associations for both financial comfort and higher physical activity of an unmeasured confounder with risk ratios of 1.16 each could suffice, but weaker confounder associations would not be enough to achieve this. Other childhood predictors, such as religious service attendance, showed slightly higher degrees of robustness to unmeasured confounders. Country-specific results can be found in the Appendix (see tables S1-S24c).

**Table 3:** Sensitivity of meta-analyzed childhood predictors to unmeasured confounding

Variable	Category	E-value for estimate	E-value for 95% CI
Relationship with mother	(Ref: Very bad/somewhat bad)		
	Very good/somewhat good	1.09	1.00
Relationship with father	(Ref: Very bad/somewhat bad)		
	Very good/somewhat good	1.11	1.00
Parent marital status	(Ref: Parents married)		
	Divorced	1.13	1.00
	Single, never married	1.20	1.00
	One or both parents had died	1.14	1.00
Subjective financial status of family growing up	(Ref: Got by)		
	Lived comfortably	1.26	1.16
	Found it difficult	1.15	1.00
	Found it very difficult	1.09	1.00
Abuse	(Ref: No)		
	Yes	1.07	1.00
Outsider growing up	(Ref: No)		
	Yes	1.10	1.00
Self-rated health growing up	(Ref: Good)		
	Excellent	1.33	1.19
	Very good	1.18	1.00
	Fair	1.17	1.00
	Poor	1.14	1.00
Immigration status	(Ref: Born in this country)		
	Born in another country	1.02	1.00
Age 12 religious service attendance	(Ref: Never)		
	At least 1/week	1.39	1.25
	1–3/month	1.33	1.19
	< 1/month	1.17	1.00
Year of birth	(Ref: 1998–2005; age 18–24)		
	1993–1998; age 25–29	1.27	1.08
	1983–1993; age 30–39	1.30	1.07
	1973–1983; age 40–49	1.33	1.00
	1963–1973; age 50–59	1.23	1.00
	1953–1963; age 60–69	1.06	1.00
	1943–1953; age 70–79	1.32	1.00
	1943 or earlier; age 80 +	1.78	1.32
Gender	(Ref: Male)		
	Female	1.67	1.51
	Other	1.92	1.39

## Discussion

Utilizing a large cross-national sample from 22 countries, we examined the impact of eleven childhood predictors as well as age and gender, on the number of days participated in physical activity during adulthood. Although the association of each predictor on physical activity in adulthood varied significantly across countries, the overall results indicated that self-rated health and religious service attendance during childhood were significantly positively associated with physical activity behavior in adulthood. In terms of gender, males were more likely to engage in physical activity than females across 22 countries, consistent with previous studies [43]. Participants' engagement in physical activity significantly decreased after their early 20s, though rose again somewhat during ages 50–69. This result is also consistent with previous studies indicating that physical activity engagement tends to peak during late adolescence and decline throughout adulthood in many countries [44–48].

Participants who reported higher self-rated health during childhood engaged in significantly more days of physical activity in adulthood. Although few studies have examined the association between perceived health status and physical activity-related behaviors, previous cross-sectional research has found that leisure-time physical activity is positively associated with self-rated health, while work-related physical activity is not associated with self-perceived health status [49, 50]. The measurement we employed to assess physical activity behavior was perhaps more likely to capture recreational physical activity rather than work-related physical activity. Previous studies have demonstrated that low levels of mental and physical health during childhood can lead to diminished health status, particularly mental health, in adulthood [51, 52]. In the present study, adulthood health status, potentially influenced by childhood health status, may have mediated the relationship between self-rated health in childhood and physical activity behavior in adulthood.

Our results also showed that the more frequent religious service attendance during childhood was positively associated with engaging in physical activity in adulthood. Previous reviews on the association between religious service attendance and physical activity behavior indicate that religiosity is positively associated with engaging in physical activity across various populations [15]. It is well-established that religiosity encompasses multiple dimensions, which may be combined in various ways [53–55]. Our measure of religiosity focuses on the behavioral dimension of religious practice, commonly described as attendance at religious services or engagement in prayer. Previous research on the trajectory of multiple dimensions of religiosity has shown that attendance at religious services is likely to be sustained from childhood into adulthood [56]. The effect of childhood religious service attendance on adult physical activity behavior may be partly mediated by the continuity of religious attendance from childhood to adulthood. In fact, attending religious services during adolescence has been linked to healthier behaviors in adulthood, such as lower smoking rates and better overall health [57]. These associations may be explained by social support and community involvement provided through religious participation. Such mechanisms may also help explain the relationship between early religious involvement and adult physical activity. One interesting finding of the present study is that more frequent religious attendance in childhood was negatively associated with days of physical activity in adulthood in a few countries, particularly in Israel. This association may be influenced by highly traditional and conservative branches of Judaism, such as Haredi Judaism, which may create conditions that restrict physical activity due to their strict adherence to Jewish law and customs.

The present study has several limitations. One of the important limitations of this study is the cultural variability in religious practices, particularly with regard to religious service attendance. For instance, in some religious traditions, such as certain branches of Buddhism or indigenous spiritual practices, regular congregational attendance is not a central practice [58, 59]. This variation in the role and frequency of religious participation could influence the interpretation of religious service attendance data and potentially introduce biases in the analysis. While we focused on attendance frequency as a behavioral indicator of religiosity, we acknowledge that it may not fully capture the diversity of spiritual engagement across different traditions. This limitation should be considered when interpreting the findings and generalizing to other religious contexts. Second, childhood predictors of physical activity were assessed retrospectively, which may introduce recall bias. Third, as with all observational research, there is the potential for confounding by unmeasured variables. For instance, the overall insignificant association between subjective financial status of the family during childhood and physical activity engagement in adulthood may be influenced by varying socio-economic levels across countries. However, for recall bias to completely explain away the observed associations would require that the effect of adult physical activity on biasing retrospective assessments of the childhood predictors would essentially have to be at least as strong as the observed associations themselves [60]. We also accounted for a range of potential confounding variables and addressed sensitivity to potential unmeasured confounding through E-value analyses, which suggested that some of the observed associations were modestly robust to unmeasured confounding. The lack of a significant association between the relationship with parents during childhood and adult physical activity behavior might be attributed to the broad nature of the concept of 'relationship' as this might be interpreted differently across different countries. More specific measurements may be necessary to obtain more meaningful results.

Despite these limitations, the present study has notable strengths. It is the first to evaluate childhood predictors of physical activity behavior in adulthood and compare results across a large number of countries (22) using large, diverse, and nationally representative samples.

## Conclusion

The results of our study highlight the potentially critical role of self-rated health and religious activity during childhood in shaping physical activity habits in adulthood across 22 countries. Our findings also help provide the foundation for utilizing future waves of data from the GFS (2024 through 2027), in order to inform the development of interventions aimed at promoting physical activity behavior among adults worldwide.

## Supplementary Information

Supplementary Material 1.

## Data Availability

Availability of data and materials: Data are available for download at the Center for Open Science (COS) website (https://www.cos.io/gfs). All analyses were pre-registered with COS prior to data access (osf.io/gam7u). Code to reproduce the analyses is openly available in the online COS repository (Padgett, Chen, et al., 2024).

## Abbreviation

GFS: Global Flourishing Study
